# Identification of the gene coding for seed cotyledon albumin SCA in the pea (Pisum L.) genome

**DOI:** 10.18699/VJGB-22-43

**Published:** 2022-07

**Authors:** A.V. Mglinets, V.S. Bogdanova, O.E. O.E. Kosterin

**Affiliations:** Institute of Cytology and Genetics of the Siberian Branch of the Russian Academy of Sciences, Novosibirsk, Russia; Institute of Cytology and Genetics of the Siberian Branch of the Russian Academy of Sciences, Novosibirsk, Russia; Institute of Cytology and Genetics of the Siberian Branch of the Russian Academy of Sciences, Novosibirsk, Russia

**Keywords:** late embryogenesis proteins, seed cotyledon albumin, peas, Pisum sativum L., CAPS marker, белки позднего эмбриогенеза, альбумины семядолей, горох, Pisum sativum L., CAPS-маркер

## Abstract

Albumins SCA and SAA are short, highly hydrophilic proteins accumulated in large quantities in the cotyledons and seed axes, respectively, of a dry pea (Pisum sativum L.) seed. SCA was earlier shown to have two allelic variants differing in mobility in polyacrylamide gel electrophoresis in acid medium. Using them, the corresponding gene SCA was mapped on Linkage Group V. This protein was used as a useful genetic and phylogeographical marker, which still required electrophoretic analysis of the protein while the DNA sequence of the corresponding SCA gene remained unknown. Based on the length, the positive charge under acidic conditions and the number of lysine residues of SCA and SAA albumins, estimated earlier electrophoretically, the data available in public databases were searched for candidates for the SCA gene among coding sequences residing in the region of the pea genome which, taking into account the synteny of the pea and Medicago truncatula genomes, corresponds to the map position of SCA. Then we sequenced them in a number of pea accessions. Concordance of the earlier electrophoretic data and sequence variation indicated the sequence Psat0s797g0160 of the reference pea genome to be the SCA gene. The sequence Psat0s797g0240 could encode a minor related albumin SA-a2, while a candidate gene for albumin SAA is still missing (as well as electrophoretic variation of both latter albumins). DNA amplif ication using original primers SCA1_3f and SCA1_3r from genomic DNA and restriction by endonuclease HindII made it possible to distinguish the SCA alleles coding for protein products with different charges without sequencing the gene. Thus, the gene encoding the highly hydrophilic albumin SCA accumulated in pea seeds, the alleles of which are useful for classif ication of pea wild relatives, has now been identif ied in the pea genome and a convenient CAPS marker has been developed on its basis.

## Abstract (ru)

Альбумины SCA и SAA – короткие гидрофильные белки, содержащиеся в высокой концентрации в семядолях и оси семени сухих семян гороха (Pisum sativum L.). Ранее показано, что альбумин SCA имеет два аллельных варианта, различающихся подвижностью в электрофорезе в полиакриламидном геле в кислой среде. С их помощью соответствующий ген SCA картирован в группе сцепления V. Белок SCA был использован как генетический и филогеографический маркер, что до сих пор предполагало проведение электрофореза белков, тогда как последовательность кодирующего гена SCA оставалась неизвестной. На основе данных, доступных в публичных репозиториях, в районе генома гороха, соответствующего позиции гена SCA на генетической карте с учетом синтении геномов гороха и люцерны, осуществлен поиск кандидатов на роль этого гена в зависимости от длины его белкового продукта, положительного заряда в кислых условиях и количества остатков лизина, ранее оцененного электрофоретическими методами. Выявленные гены просеквенированы у ряда образцов гороха. Соответствие полученных электрофоретических данных и нуклеотидной изменчивости позволило идентифицировать последовательность Psat0s797g0160 из референсного генома гороха в качестве гена SCA. Последовательность Psat0s797g0240, возможно, кодирует родственный минорный альбумин SA-a2, тогда как ген-кандидат альбумина SAA остается неидентифицированным (как и электрофоретическая изменчивость двух белков, упомянутых последними). Амплификация ДНК с использованием оригинальных праймеров SCA1_3f и SCA1_3r и геномной ДНК в качестве матрицы, а также расщепление ее эндонуклеазой рестрикции HindII позволяют различать аллели гена SCA, белковые продукты которых имеют разный заряд, без секвенирования. Таким образом, ген, кодирующий высокогидрофильный альбумин SCA, накапливающийся в семядолях гороха, аллели которого полезны для классификации диких родственников культурного гороха, идентифицирован в геноме гороха, и на его основе разработан удобный CAPS-маркер.

## Introduction

Mature pea (Pisum sativum L.) seeds contain a large amount of protein families, which are generally classified as globulins (soluble in salt solutions) and albumins (soluble in water), the former being mostly storage proteins, the latter having a number of functions, including substitution of water in dry tissues (Smirnova et al., 1990). A number of albumins are extractable from seed flour with 5 % perchloric acid, which were characterised in detail by O.G. Smirnova et al. (1990, 1992). Special attention was drawn to the two most abundant of these albumins, which are biochemically and immunologically related and quite short (about 100 amino acid residues) highly hydrophilic peptides. One of them predominates in the cotyledons and the other in the seed axis, both accumulating during seed formation and depleting during germination. They were respectively named SCA (seed cotyledon albumin) and SAA (seed axis albumin). Their amino acid content and the accumulation pattern in seeds left no doubt as to their participation in substitution of water in dry seed cells, that is in a dehydrin-like function, although they differ in many respects from the known dehydrins and are much smaller proteins (Smirnova et al., 1992). While the SAA protein was electrophoretically monomorphic in peas, two allelic variants of SCA were revealed to differ in electrophoretic mobility in 15 % polyacrylamide gels containing acetic acid and urea according to S. Panyim and R. Chalkley (1969), which allowed to genetically map the relevant gene SCA (Smirnova et al., 1992; Rozov et al., 1993; Gorel et al., 1998) on linkage group V (corresponding to chromosome 3; Smýkal et al., 2012; Kreplak et al., 2019).

O.G. Smirnova et al. (1992) found out that the fast electromorph SCAf was frequent in the wild subspecies of the common pea (P. sativum subsp. elatius Aschers. et Graebn.) but was extremely rare in the cultivated subspecies P. sativum L. subsp. sativum. (Here, the inclusive taxonomic system of peas according to N. Maxted and M. Ambrose (2001) is followed.) O.E. Kosterin and V.S. Bogdanova (2008) and O.E. Kosterin et al. (2010) noticed a strong concordance of the occurrence of SCAf with that of the plastid rbcL allele containing a recognition site for the Hsp AI restriction endonuclease, and a less strong concordance with that of the mitochondrial cox1 allele containing the recognition site for the Psi I restriction endonuclease. This concordance was interpreted in terms of the common phyletic origin and as evidence of the existence of two different wild pea lineages. Based on this, different combinations (A, B and C) of alleles of the three mentioned dimorphic marker genes SCA, rbcL and cox1 from different cellular genomes, respectively nuclear, plastid and mitochondrial, were proposed for a simple classification of evolutionary lineages of the wild pea subspecies P. sativum subsp. elatius (Kosterin, Bogdanova, 2008; Kosterin et al., 2010), which was then used repeatedly (Zaytseva et al., 2012, 2015, 2017; Kosterin, Bogdanova, 2021; Bogdanova et al., 2021). So, the electromorphs of SCA appeared to be useful in the studies of genetic diversity of the pea crop wild relatives, which are important for the involvement of their potentially useful genetic resources into breeding (Ali et al., 1994; Maxted, Kell, 2009; Coyne et al., 2011; Ford-Lloyd et al., 2011; Maxted et al., 2012). However, while the plastidic and mitochondrial markers were scored by the CAPS approach involving DNA amplification and restriction, the SCA gene sequence remained unknown and analysis of this marker required more laborious protein electrophoresis. Molecular identification of this gene would be desirable to facilitate the analysis. This became possible when the pea nuclear genome was published by K. Kreplak et al. (2019). This communication is devoted to identification of the SCA gene in the pea genome, its brief characterisation and working out a convenient CAPS marker based on this gene.

## Materials and methods

The gene SCA was sequenced from samples of DNA extracted in the course of our previous work (Kosterin, Bogdanova, 2008), from the following pea germplasm accessions: 721 (Israel), CE1 (Crimea), CE11 (= JI3557, Portugal), JI1794 (Golan Heights), L100 (Israel), PI344538, Pse001, Pe013, P015, P017 (Turkey), VIR320’ (Palestine) (P. sativum subsp. elatius), VIR3429 (Egypt), VIR4911 (Tibet), VIR5414 (Ethiopia), VIR7335 (Tajikistan), WL1238 (a testerline), cultivar Cameor (P. sativum subsp. sativum), VIR4871 (Pisum sativum subsp. transcaucasicum Govorov), VIR2759 (Ethiopia) (Pisum abyssinicum A. Br.), and WL2140 (Israel) (P. fulvum Sibth. et Smith). VIR accessions were received from N.I. Vavilov All- Russian Institute of Plant Genetic Resources, Saint-Petersburg, accessions 721, Pse001, Pe013, P15, P17, JI1794, L100 and Cameor were kindly provided by Dr. Norman Weeden, Cornell University, New York, accession PI344538 was kindly provided by Dr. Petr Smýkal, Olomouc University, the progenitors of accessions CE1 and CE11 were collected in nature by the third author. Polymerase chain reaction to amplify the fragment corresponding to Psat0s797g0160 was carried out using BIS 208 cycler in 20 μl of the PCR reaction mixture under a mineral oil layer with the following cycling parameters: 95 °C for 3 min; 45 cycles consisting of denaturation at 94 °C for 30 s, annealing at 56 °C for 25 s and elongation at 72 °C for 40 s; final elongation at 72 °C for 5 min; for amplification of the fragment corresponding to Psat0s797g0240 the same parameters were used but annealing was at 59 °C. For restriction analysis, 5 μl of the resulting reaction mixture were digested with 1 unit of HindII endonuclease according to manufacturer’s instructions and the products analysed on 1.5 % agarose gel in TAE buffer. The 100-bp ladder (SibEn zyme, Novosibirsk) was used as a molecular mass marker. For sequencing, PCR products were purified by 20 % polyethylene glycol 800 in 2.5 M NaCl. Sanger reaction was carried out using BrightDye Terminator version 3-100 (Nimagen, Netherlands) with the conditions recommended by the manufacturer for 50 cycles. The Sanger reaction products were purified using Sephadex G-75. Sequencing was carried out in Genomic Core Facility SB RAS, Novosibirsk. The sequences obtained in this study were submitted to European Nucleotide Archive with the following entry numbers OU953856- OU953865, OU953869- OU953881 for SCA and OU953866- OU953868, OU953882- OU953894 for alleles of Psat0s797g0240.

## Results and discussion

Candidate gene for SCA seed albumin in public databases The SCA gene is mapped on likage group V (LGV) between the loci His1 (coding for histone H1 subtype 1) and coch (cochleata) (Gorel et al., 1998) (its earlier published position behind coch (Rozov et al., 1993) was tentative as based on non-additive data with respect to these three loci). The potential candidates were searched in the annotated genome of Medicago truncatula Gaertn. making use of its synteny with the genome of Pisum (Kalo et al., 2004). The bordering markers coch and His1 of pea correspond to the loci with Gene IDs 11417633 and 25499208, respectively, therefore suitable candidates found in Medicago truncatula should map to chromosome 7, syntenic to LGV of P. sativum (Kalo et al., 2004; Kreplak et al., 2019) at physical position between 42,203,622 (GeneID: 11417633) and 45,488,994 (GeneID: 25499208) on NC_053048.1 (M. truncatula strain A17 chromosome 7). This region contained 421 coding sequences, of which three neighbouring loci were annotated as “18 kDa seed maturation protein”. Two of them, LOC11421661 and LOC11437338, encoded polypeptides of 105 and 101 amino acids, respectively. The third locus, LOC11437936 encoded polypeptide of 177 amino acids. These polypeptides were used as a query to search the P. sativum genome assembly at https://urgi.versailles.inra.fr/blast. All three searches retrieved the same hits, Psat0s797g0240 and Psat0s797g0160, separated by about 25 Kb on the scaffold 00797 not attributed to any chromosome and Psat3g068920 on chr3LG5 with physical position between the loci coch and His1. Psat3g068920 encoded polypeptide of 190 amino acids and probably corresponded to the LOC11437936 of M. truncatula while Psat0s797g0240 and Psat0s797g0160 probably corresponded to LOC11421661 and LOC11437338. Both encoded polypeptides of 101 amino acids and were concluded to be ideal candidates to represent the SCA locus.

Psat0s797g0160 with position 57,488–57,939 on scaffold00797, annotated as “Late embryogenesis abundant (LEA) group 1”, encoded a polypeptide of 101 amino acid residues including 18 positively charged residues, of which 10 were lysines. Psat0s797g0240 with position 83,885–84,190 on scaffold00797, also annotated as “Late embryogenesis abundant (LEA) group 1”, encoded a polypeptide of 101 residues including 18 positively charged residues, of which 11 were lysines. The earlier obtained data on amino acid composition of the SCA protein were as follows. The slow electromorph SCAs, common in cultivated peas, was estimated by the incomplete succinylation method to possess 17 positively charged residues, including 9 lysines, and, together with data on the amino acid content, as being ca 107 residues long (Smirnova et al., 1992). This was a rather good correspondence of data from protein chemistry and sequencing. The estimate of 9 rather than 10 lysine residues in SCAs with the incomplete succinylation method (Smirnova et al., 1992) may be explained by a tandem of two lysine residues in positions 47–48 of the deduced protein product, which could not both bind to succinic acid residues for steric reasons.

Hence the size and amino acid content of the protein products and genome location, as concluded from the synteny with M. truncatula, of both Psat0s797g0160 and Psat0s797g0240 corresponded to SCA (Smirnova et al., 1992; Gorel et al., 1998). To choose between them for a candidate for SCA we made use of the genetic data. SCA was shown to be dimorphic with allelic variants differing with respect to the number of positively charged amino acid residues while SAA was monomorphic in this respect (Smirnova et al., 1992). Sequences available in sequence read archives (SRA) at NCBI containing data of high throughput resequencing of pea accessions were used to assemble alleles of Psat0s797g0160 and Psat0s797g0240 from pea accessions W6_2107 (BioProject PRJNA431567), JI1794, WL2140 (Pisum fulvum Sibth. et Smith) (BioProject PRJNA431567), JI2202 (Pisum abyssinicum A.Br.) (BioProject PRJNA285605), 711 (BioProject PRJEB30482), 721 (BioProject PRJNA431567, PRJEB30482). Five of these accessions were involved in our previous electrophoretic studies of SCA (Kosterin, Bogdanova, 2008); Cameor and JI1794 were shown to have the slow electromorph SCAs while WL2140, 711 and 721 had the fast electromorph SCAf. There was some sequence variability among alleles of Psat0s797g0160. Two nucleotide substitutions differed in the alleles of Cameor, W6_2107 and JI1794 from those of WL2140, JI2202, 711, 721, namely T/G in the position 215 (from start codon) and A/C in the position 238. The T/G substitution changed valine for glycine, and the A/C substitution changed asparagine to histidine, which is positively charged under conditions of acetic acid-urea PAAGE used for SCA analysis. Thus, the latter amino acid replacement affects electrophoretic mobility of the encoded protein and is associated with the fast electromorph. Allelic variants of Psat0s797g0240 did not carry amino acid substitutions associated with the change of electrophoretic mobility. This allowed us to nominate Psat0s797g0160 as a candidate for the SCA gene.

Concordance of the sequence variation of the candidate gene for SCA with SCA electrophoretic pattern

To confirm Psat0s797g0160 to be the SCA gene we resequenced it in 20 pea accessions in which SCA was previously studied electrophoretically (Smirnova et al., 1992; Kosterin, Bogdanova, 2008; Kosterin et al., 2010). To design primers matching 3′ and 5′ non-coding regions we searched public databases for pea sequences coding for the same protein as Psat0s797g0160. The search retrieved the sequence PUCA013656022.1 (Pisum sativum, cultivar Gradus No 2 flattened_line_3009, whole genome shotgun sequence) containing the coding sequence as well as 5′ and 3′ non-coding regions. The primers Ps_SCA1_3f (5′ GCAT CATACTCTTCAACACAT) and Ps_SCA1_3r (5′ GTAG GAACATTCACAACATCA) were designed to match those non-coding regions. We sequenced the coding region of the SCA gene in two groups of 10 pea accessions each, including: (i) 711, 721, VIR320’, CE11, PI344538, Pe013 (P. sativum subsp. elatius), VIR2759 (Pisum abyssinicum A.B.), VIR3429, VIR7335 (P. sativum subsp. sativum) and WL2140 (P. fulvum) which were shown (Kosterin and Bogdanova, 2008) to have the fast SCA electromorph (SCAf ), frequent in wild peas, and (ii) accessions CE1, JI1794, Pse001, P015, P017 (P. sativum subsp. elatius), VIR4871 (Pisum sativum subsp. transcaucasicum), VIR4911, VIR5414, WL1238 and Cameor (P. sativum subsp. sativum) which were shown to have the slow SCA electromorph (SCAs), predominating overwhelmingly in the cultivated pea but occurring in wild peas as well. The derived amino acid sequences had 19 and 18 positively charged amino acid residues in the first and second group, respectively. Full correspondence of the electrophoretic and sequence data, together with the genomic position and the size and content of the protein product, indicate that Psat0s797g0160 is the SCA gene (the latter designation will be used in the text below).

The related gene Psat0s797g0240

The gene Psat0s797g0240 resides in the pea genome (cultivar Cameor) in about 25 Kb from Psat0s797g0160, the two loci have very similar sequence, and the difference in their coding sequences is 25 nucleotides (8.2 %). Obviously, these genes are paralogs originated by tandem duplication of a genome region. The inferred amino acid sequence of the Psat0s797g0240 polypeptide product has the same length of 101 amino acid sequences and differs (in cv. Cameor) by eight amino acid substitutions from that of Psat0s797g0160 (their positions are given in parenthesis): ala→ser (14), arg→lys (32), tre→ala (35), arg→his (36), tre→ser (70), val→gln (72), gln→arg (79), asn→his (80). Due to the two latter substitutions this polypeptide has two positively charged residues more than SCA. The mobility of polypeptides of equal length in the involved electrophoretic procedure in acid denaturing conditions is proportional to the number of positively charged residues, so we can expect the Psat0s797g0240 product mobility to be 11 % greater than that of SCAs. However, the SAA mobility is only 7 % greater than that of SCAs (Smirnova et al., 1992). This is close with the mobility of SCAf, which is 5 % greater than that of SCAs (Smirnova et al., 1992), as expected from their difference of one positively charged residue. Most probably, Psat0s797g0240 is not the gene coding for SAA but may encode an immunologically related minor protein SA-a2 with electrophoretic mobility 9 % greater than in SCAs. Electrophoretic monomorphism of both SAA and SA-a2 so far observed (Smirnova et al., 1992) does not allow us to check these options genetically. We attempted resequencing the Psat0s797g0240 alleles in the same set of accessions as for SCA (see above) using primers Ps_SCA2_1F (5′ CACGTGTTCAATAATCTAACGC) and Ps_SCA2_1R (5′ AAGAAAAAGAAACGAGCCATCA) matching the 5′- and 3′ non-coding regions of PUCA011001169.1 (Pisum sativum cultivar Gradus No 2 flattened_line_64181, the whole genome shotgun sequence). Possibly, due to the abundance of poly-A and poly-T in the 5′- and 3′ non-coding regions, respectively, amplification was not successful for 7 of 20 accessions involved (JI1794, 721, Pe013, P014, P017, VIR5414, WL1238, Cameor), so only 13 accessions were sequenced. The variation of protein products inferred from the obtained sequences was confined to 5 variable amino acid positions and did not affect electrophoretic mobility.

Protein product of SCA and its variation

The SCA gene has no introns. Its SCA protein product is remarkable for its extreme hydrophily and high content of charged (at neutral pH conditions) residues (Table). Among its 101 amino acid residues in Cameor, 70 are hydrophilic, of which 30 are charged, including 18 positively charged (lysine – 10, arginine – 5, histidine – 3), and 12 negatively charged (glutamate – 10, aspartate – 2) residues (the numbers of residues almost coinciding with their percentages) (see Table). Interestingly, 11 of 12 negatively charged residues have positively charged nearest neighbour(s) and 4 of 18 positively charged residues have negatively charged neighbour(s). There are tracts of three (glu-lys-glu) and five (lys-lys-glu-glu-arg) charged residues in a row. There are only two proline residues and only one aromatic residue (tyrosine). Such a content and structure, with alternating residues of opposite charge, suggest that in water solution, the SCA molecule has a rigid expanded (linear) structure

**Table 1.**
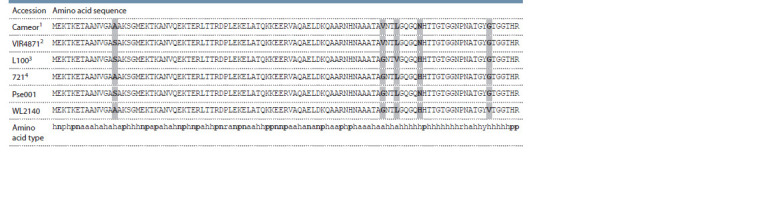
Variants of the deduced amino acid sequences encoded by SCA alleles sequenced from pea accessions (polymorphic positions boldfaced and highlighted) Note. The lowest line shows amino acid types encoded as follows: a – aliphatic, h – hydrophilic, n – negatively charged in neutral conditions, p – positively charged in neutral conditions, r – proline, y – aromatic (tyrosine) (charged types boldfaced). 1 the same in WL1238, VIR4911, VIR_5414, JI1794, CE1, P017, W6_10925; 2 the same in P015; 3 the same in VIR320’, VIR2759, VIR3429, VIR7335; 4 the same in W6_26109, Pe013, CE11, PI344009, PI344538

Five variable amino acid positions were revealed in the SCA product: 14 (ala/ser), 72 (gly/val), 75 (leu/val), 80 (his/asn) and 95 (gly/val) (see Table), the fourth changing the molecule positive charge as discussed above. The SCA gene sequences obtained had 11 (3.6 %) variable nucleotide positions.

SCA gene and a CAPS marker based on it

The A→C substitution in position 238 of the SCA gene creates the recognition site GTCAAC for HindII restriction endonuclease, missing in the rest of the gene. PCR amplification of genomic DNA with Ps_SCA1_3f and Ps_SCA1_3r primers resulted in a product of 512 bp, which was subsequently digested with HindII endonuclease. The allele coding for SCAf was cleaved into two fragments, 326 and 186 bp in size, while amplicon from the SCAs allele remained 512 bp, the difference clearly seen on agarose gel (Figure). This makes the SCA gene the source of a convenient CAPS (cleaved amplified polymorphic sequence) marker, which permits scoring SCA allelic state without invoking protein electrophoresis. It should be noted that in spite of great similarity of the coding sequences of SCA and Psat0s797g0240, their adjacent non-coding regions appeared diverged enough to avoid cross-amplification.

**Fig. 1.**
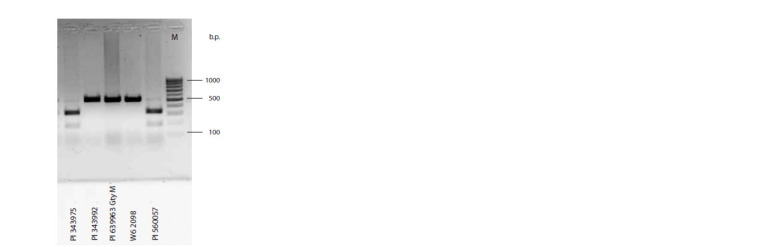
An example of agarose gel electrophoresis of the PCR products obtained from genomic DNA of the indicated pea accessions with primers Ps_SCA1_3 and Ps_SCA1_3r, matching the SCA gene adjacent non-coding regions, digested with HindII restriction endonuclease. M stands for the molecular mass marker.

## Conflict of interest

The authors declare no conflict of interest.
